# Assessment of modifications to evidence-based psychotherapies using administrative and chart note data from the US department of veterans affairs health care system

**DOI:** 10.3389/fpubh.2022.984505

**Published:** 2022-11-15

**Authors:** Shannon Wiltsey Stirman, Heidi La Bash, David Nelson, Robert Orazem, Abigail Klein, Nina A. Sayer

**Affiliations:** ^1^National Center for PTSD, VA Palo Alto Healthcare System, Menlo Park, CA, United States; ^2^Department of Psychiatry and Behavioral Sciences, Stanford University School of Medicine, Stanford, CA, United States; ^3^Center for Care Delivery and Outcomes Research, Minneapolis VA Health Care System, Minneapolis, MN, United States; ^4^Department of Medicine, University of Minnesota, Minneapolis, MN, United States; ^5^Department of Medicine and Psychiatry, University of Minnesota, Minneapolis, MN, United States

**Keywords:** implementation, evaluation, adaptations, modifications, fidelity

## Abstract

**Background:**

The US Department of Veterans Affairs (VA) has over 15 years of experience in delivery of evidence-based psychotherapies (EBPs). This paper describes strategies for using clinical documentation and administrative data to understand adherence and modifications to EBPs for Posttraumatic Stress Disorder (PTSD).

**Methods:**

This study focused on two EBPs for PTSD, Cognitive Processing Therapy (CPT) and Prolonged Exposure (PE). The sample included VA therapists from across the US who provided CPT and PE and the patients they treated over a 1-year period. The data sources for this study were templated EBP chart notes and VA administrative data. We used a manual review of note content and administrative data rules to code therapy adherence and modifications in 7,297 EBP sessions for 1,257 patients seen by 182 therapists. Two trained coders rated each therapy note and resolved discrepancies through consensus. To contextualize and explain variation in adherence and modifications, we conducted brief 30–45-min semi-structured interviews with a purposive subsample of these therapists (*n* = 32).

**Findings:**

Combining manual chart review and administrative data allowed for identification of 11 types of modifications. Raters disagreed on adherence for 30% of notes. The disagreement stemmed from the presence of therapy modifications that were not clearly documented, necessitating the development of decision rules and strategies for modification coding. Both therapists and patients contributed to the variance in the extent to which different modifications occurred. Therapist interviews demonstrated therapist awareness of modifying the protocols in the ways identified through chart review.

**Conclusion:**

Healthcare systems can use data collected as part of routine care to understand how and when EBPs are modified but need to develop scalable strategies to document adaptations and modifications to EBPs in routine care.

## Introduction

As evidence-based treatments have been implemented into routine care treatment settings, there have been questions about the appropriate balance between fidelity and modification. When large-scale implementation efforts began in many systems, fidelity (comprising adherence, or the provision of all unique and essential components of the treatment, and competence, or skill with which those elements are provided (1)) was a primary training goal. However, it has been increasingly clear that implementation in routine care frequently includes modifications (defined as any changes to an intervention that deviates from the originally specified materials or processes) to different aspects of the treatments, either in the form of adaptations (planned, intentional and ideally data-driven modifications made to address a need, or constraint) or unplanned, often improvised changes (1). At times, modifications or adaptations are consistent with the protocol and do not compromise fidelity, whereas at other times modifications may lead to reduced exposure to effective elements of the treatments.

Modifications of interventions for mental health have been documented in a variety of treatment settings (2–4). For example, in a study of residential programs for PTSD in the United States (US) Veterans Health Administration's healthcare system (VA), therapists reported that they made numerous types of changes to two evidence-based psychotherapies (EBPs) for PTSD, Cognitive Processing Therapy (CPT) and Prolonged Exposure [PE; (5)]. In a chart review of 131 veterans who received one of three EBPs for PTSD in a VA PTSD specialty care setting, 62% of the veterans experienced at least one protocol modification over the course of the episode of care (6). Therapists report making modifications in an effort to address factors such as comorbidity, cultural norms, language and literacy differences, and contextual constraints (5, 7, 8). Understanding the types of adaptations and modifications that are made, especially in conjunction with fidelity and effectiveness data, can facilitate efforts to improve the fit, reach, and effectiveness of interventions in routine care settings. For example, in a study involving observation-based coding of modifications over the full treatment protocol, Marques et al. found that in a sample of Spanish- and English-speaking consumers in a community mental health setting, both fidelity and modifications that remained consistent with the CPT protocol were associated with increased treatment effectiveness (9). In another study, Yu et al. found that two therapist-described adaptations (extending of the protocol and modification of content) were associated with the extensiveness with which protocol elements were covered in youth mental health settings (10). High quality documentation is necessary to advance efforts to understand the types of modifications that are made and the impact that they have on both clinical and implementation outcomes (1).

A variety of methods can be used to identify adaptations and modifications to EBPs, including observation, therapist interviews, self-report, and medical record/clinical documentation review. Each has advantages and drawbacks in terms of resources required, reliability, and level of detail (1, 11). Using clinical documentation from medical records can be advantageous because it can minimize the additional burden to clinicians beyond completion of required clinical notes and documentation. However, to our knowledge, this approach has only been used in one mental health-related study to date, within a single clinic (6). In this paper, we describe an approach that involves human and electronic coding of session-level templated clinical notes in VA medical records for both modifications and adherence. We describe the coding processes, report findings on modifications that were identified using this method, explore therapist and patient contribution to modification use, and make recommendations for the use of clinical documentation to identify modifications. We hypothesized that: (1) Treatment modifications would be common; (2) There would be systematic differences between therapist and patient contributions to session-level modifications; and (3) Human coders would have more difficulty coding sessions for adherence and modifications when therapists' adherence was low.

## Methods

**Figure 1:**
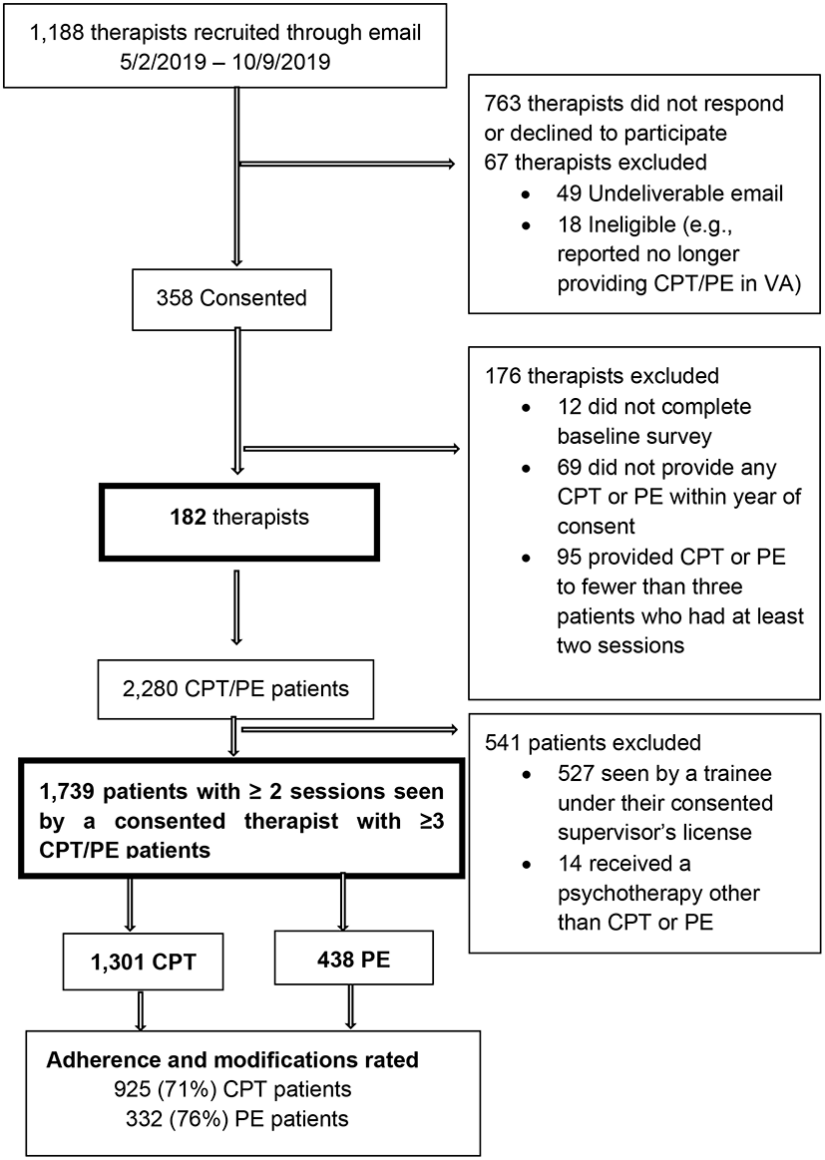
Flow of participants for adherence and modifications rating.

Data for this study was drawn from a larger project that was approved by the local Institutional Review Board. Therapist enrollment took place between May 2, 2019 and October 9, 2019. We extracted clinical notes for patients to ensure that there was at least 6 months of data following the first session for all CPT and PE patients of enrolled therapists. Figure 1 shows the flow of therapists and patients into the study.

To obtain a representative sample of therapists, we stratified the full population of 2,962 licensed VA mental health professionals who provided individual CPT or PE to at least two patients in 2018 into 12 strata, based on type of EBP they provided (individual CPT, individual PE, or both) and US geographic region (West, South, Midwest, Northeast). We used the proportions of the 2,962 in each stratum to identify the target proportional sample size for each stratum. While our target analytic sample size was ~200 therapists, our target sample for recruitment was 350 therapists to allow for the possibility that therapists who enrolled might leave VA or change employment within VA or provide CPT and/or PE to fewer than three patients during the study period. This paper focuses on documentation from the 182 therapists and 1,257 patients whose chart notes were reviewed for adherence and modifications.

We excluded therapists who could not be contacted through VA email, because they were presumably no longer working for the VA, and those who emailed the study team to state that they were no longer providing CPT or PE due to a change in job responsibilities. The Institutional Review Board-approved online consent process explained the purpose of the study and that therapist participation involved: (1) completing an online 15-min survey about their work environment, (2) watching a 5-min refresher tutorial that summarized EBP documentation requirements using CPT and PE templates, and (3) the study team extracting information about their use of CPT and/or PE from their patients' medical records. Among those who consented, we excluded therapists who did not complete a provider survey used for the main study and those who saw fewer than three CPT and/or PE patients who participated in at least two sessions within the year of therapist consent.

We used VA EBP templates in the medical records to prospectively identify the patients who began a course of individual CPT or PE with the consented therapists within the year of therapist consent. The Institutional Review Board granted a waiver of informed consent for patients, as we were monitoring routine care through existing medical records. We included those patients diagnosed with PTSD who had at least two CPT or PE individual therapy sessions. We used manual chart note review to exclude: (1) patients seen by unlicensed mental health professionals (e.g., psychology interns and other trainees) working under the consented licensed professional, and (2) patients who received psychotherapies other than CPT or PE even though those sessions were documented with a CPT or PE template. The decision to remove patients seen by trainees enabled us to link patients to independently licensed professionals and to reduce error estimates as we examined variation in outcomes attributable to therapists as part of the parent study. Of the 2,280 patients who met inclusion criteria, we excluded 527 because they were seen by a trainee under their consented supervisor's license and 14 because they were receiving a psychotherapy other than CPT or PE.

### Medical record data

Both adherence and modifications were identified through medical records. The templates include text identifying EBP type (CPT vs. PE) and check boxes for the unique and essential elements of each session for each protocol, as well as free text boxes. Before signing the notes, therapists can remove, modify, add, or delete the text that is automatically generated by checking boxes on the template. For CPT, there is a unique template for each of the 12 sessions from the CPT protocol. For PE, there is a unique template for each of the first 3 sessions, sessions 4–12, and the final session. Since PE session 2 covers a lot of material, the PE template includes a method for indicating whether the content was split across 2 sessions. The PE template for session 4–12 is designed to be used for multiple sessions, depending on the number of exposure sessions needed. The raters also rated adherence from the free text of untemplated CPT and PE notes that were clearly CPT or PE notes as demonstrated by the listing of the content included in the templated notes. To ensure that we had data on adherence for an adequate number of sessions covering different essential elements for each EBP for an adequate number of patients, we planned to manually rate up to the first 7 sessions for up to 10 patients per therapist. However, we rated more patients for some therapists included in our training set. When the templated notes revealed a break in the sequencing of sessions (e.g., we found templates for CPT sessions 3 and 5 but not session 4), we reviewed chart notes that were not templated and rated adherence and modifications in any identified untemplated CPT and PE notes for that patient. The vast majority (95.3%) of the CPT and PE sessions for the included therapists were templated. We rated a total of 7,297 sessions for 1,257 (72.3%) of the 1,739 patients seen by the therapists in our sample.

### Coding process

The rating team included four trained raters. Rater training included joint review of notes for 106 randomly selected EBP patients of the enrolled therapists. The first author provided training in assessing modifications, helped with development of the modifications codebook, and provided consultation as needed. Expert CPT and PE clinicians who were also familiar with the template and adherence and modification ratings joined meetings to help resolve discrepancies.

Raters were randomly assigned to patients rather than notes so they could get a full picture of the progression of each patient's therapy course. The coding process involved multiple steps. First, raters reviewed each patient's notes to verify that the patient was receiving individual CPT or PE delivered by a licensed therapist and not a trainee. Next, raters double coded the note elements to determine what session from the respective protocols (i.e., protocol session number) was being delivered. For training and calibration purposes, we began with complete double coding of all sessions. Complete double coding involved double coding of the session number and individual items within a session. We checked agreement on session number and items between raters in batches. Through this process, we determined that when raters agreed on the protocol session number that the therapist was delivering (e.g., content from CPT protocol session 3 was covered), agreement on the individual session components was excellent−95.5% for CPT and 95% for PE. Therefore, when two raters agreed on session number, we had a single rater code the remaining session components. This occurred for approximately one-third of the sample. Hereafter we refer to this process as Double-Single Coding, to reflect that session number was always double coded, but session elements were coded by one rater. We implemented coding of both session number and items by two raters (hereafter called Double Coding) when raters could not agree on session number. Double Coding was also used on a subset of all records to ensure ongoing calibration. Consensus Coding was used if one rater requested that two raters code a session together because they found the documentation to be particularly confusing or if the agreement check found that two raters did not agree on the protocol session number. Throughout the rating process, the raters met weekly to review cases and resolved discrepancies through consensus. The raters also maintained a codebook documenting the decision rules and the challenges that led to the need for Consensus coding as well as to track decision rules. Two authors who were also raters (RO, AK) reviewed the documentation of coding challenges and grouped them into themes.

### Therapy adherence

Raters coded the unique and essential elements that were endorsed in the templated medical records. The unique and essential items were based on the adherence forms used for a large CPT and PE comparative effectiveness study (12). For CPT, raters indicated whether the patient received CPT with or without the optional trauma account at the session level. We calculated adherence scores for each completed session as the number of the unique and essential items present for that session out of the total number of unique and essential items for that session included in the template. If a therapist skipped a protocol session (e.g., provided content from sessions 1, 2, 4, 5, but skipped 3), the removed session was scored 0% adherence. When a therapist repeated a session (e.g., provided session 2 content in two separate sessions), we combined the unique and essential elements documented across sequentially repeated sessions.

### Therapy modifications

We used the Framework for Reporting Adaptations and Modifications—Expanded (FRAME; 1) to code EBP modifications. The FRAME was developed to identify the following nine types of content modifications: tailoring/tweaking, integrating another treatment (e.g., mindfulness), session lengthening, protocol lengthening, session shortening, re-ordering, repeating, spreading content over multiple sessions, and drift. Table 1 contains the operational definitions of the modifications used and how they were identified in the medical records. While coding adherence, the raters assigned ratings for tailoring, integration of another treatment, and drift. *Removing* was identified as present when adherence was <100%. We did not include protocol shortening, because it was not reliably distinguishable from patient-initiated dropout, based on the information available in the medical records. Raters also extracted the recorded number of minutes for each session to determine whether sessions were shortened or lengthened. We categorized CPT therapy courses completed in more than 12 sessions and PE therapy courses completed in more than 15 sessions as protocol lengthening.

**Table 1:** Modifications identified in templated cognitive processing and prolonged exposure notes.

			**Proportion with documented modification**			**Rating strategy**			
**Modification type**	**Definition**	**Data source**	**Therapists** ***N* = 182** ***N* (%)**	**Patients ** ***N* = 1,257** ***N* (%)**	**Sessions** ***N* = 7,297** ***N* (%)**	**Double -single coding**	**Double coding**	**Consensus coding**	**Comparison**
						***N* (%)**	***N* (%)**	***N* (%)**	**OR (CI), *p***
Tailoring/Tweaking	Clinician changes the packaging of the EBP but intervention content is intact. Example: Modifying homework format/structure; including family member in the session	Determined by raters based on documentation	88 (48%)	131 (10%)	183 (3%)	40 (9%)	40 (11%)	51 (12%)	DS vs. D .82 (0.52-1.30), .40 D vs. C 0.875 (0.56–1.36), 0.55 DS vs. C 0.72 (0.47–1.11), 0.14
Removing	Therapist's documentation shows that they left out unique and essential elements associated with a given session.	Scored when raters determined that adherence for a given session, whether delivered once or repeated was < 100%.	180 (99%)	966 (77%)	2,209/6,512 sessions that were not repeated (34%)	-	-	-	-
Switching CPT type	Therapist documents that they are starting with either CPT with or without account but then switch to the other CPT type.	Determined by raters based on documentation in notes of CPT therapists (*N* = 165)	37 (22%) 9 (5%) among therapists providing CPT switched > 1x	46 among 925 patients receiving CPT (5%)	57 (1%) among 5,422 CPT sessions	-	-	-	-
Integrating another treatment	While the EBP is the starting point the clinician also uses a different therapeutic approach. Example: *In vivo* or exposure during CPT; use of mindfulness skills training.	Determined by raters based on documentation. Not used for drift (see below)	34 (19%)	40 (3%)	45 (< 1%)	-	-	-	-
Session lengthening/extending	The clinician spends a longer amount of time than prescribed to complete a session.	Number minutes in note extracted by raters For CPT > 60 min For PE > 90 min	105 (58%)	246 (20%)	529 (7%)	92 (20%)	51 (13%)	103 (24%)	D vs. DS 1.64 (1.12–2.39) 0.0092 C vs. D 0.49 (0.34–0.71), < 0.0002 C vs. DS 0.81 (0.59–1.11), 0.19
Protocol lengthening/extending among protocol completers	The clinician uses more sessions than prescribed to complete the treatment.	Count of total number of EBP sessions For CPT > 12 sessions For PE > 15 sessions	33 (20%)	54 (10%) among 535 patients completing EBT	NA				
Session shortening/condensing	The clinician spends a shorter amount of time than prescribed to complete sessions. Example: Clinician covers prescribed elements from more than one protocol session (e.g., session 2 and 3) during same 60-minute appointment or appointment is briefer than prescribed.	Number minutes in note extracted by raters For CPT < 45 min For PE < 60 min	171 (94%)	604 (48%)	1,919 (26%)	152 (34%)	266 (70%)	186 (43%)	D vs. DS .21 (0.159–0.287), < 0001 C vs. D 3.09 (2.31–4.13), < 0.0001 C vs. SD 0.66 (0.50–0.87), .003
Repeating	Session prescribed once during a protocol is delivered more than once. Clinician may not explicitly write that they are repeating but it is clear from the content that a prior session is repeated. Repeated sessions may not be consecutive.	Protocol number raters assign to the session based on documentation of prescribed elements is repeated.	147 (81%)	466 (37%)	787 (11%)	124 (27%)	95 (25%)	247 (58%)	D vs. DS 1.13 (0.83–1.54), 0.44 C vs. D 0.25 (0.18–0.33), < 0.0001 C vs. DS 0.28 (0.21–0.37), < 0.0001
Reordering	Adjusting the order of intervention modules or segments. Example: session 6 CPT delivered before session 5.	Order of protocol number raters assign to the session based on documentation of prescribed elements is not consecutive. Not used for consecutive repetition of sessions.	4 (2%)	4 (0%)	8 (< 1%)	0	0	4 (< 1%)	n/a
Spreading	Breaking up session content over multiple consecutive sessions. Example: Some of protocol session 3 is covered in first instance of session 3 and the rest is covered in second instance of session 3 the following week.	Protocol number raters assign is the same as a prior session but covers different prescribed content. Not coded for PE session 2 divided into 2a and 2b because this spreading is specified in PE protocol	129 (71%)	317 (25%)	554 (8%)	88 (20%)	62 (16%)	167 (39%)	D vs. DS 1.24 (0.87–1.77), 0.25 C vs. D 0.31 (0.22–0.43), < 0.0001 C vs. DS 0.38 (0.28–0.51), < 0.0001
Drift	It is clear from the note that patient experiences are discussed outside of the structure of the EBP or a templated note includes no prescribed elements. Example: CPT note in which patient life event discussed extensively without identification of stuck points or use of worksheet.	Determined by raters based on documentation	103 (57%)	183 (15%)	229 (3%)	38 (8%)	50 (13%)	95 (22%)	D vs. DS 0.60 (0.39–0.94), 0.027 C vs. DS 0.53 (0.37–0.78), 0.001 C vs. DS 0.32 (0.22–0.48), < 0.0001

All modifications except protocol extending were based on EBP sessions 1 through 7. Protocol extending based on total number sessions. Removing was not analyzed because it was the inverse of adherence.

Double-Single: Double coding of session number/Single coding of items when agreement on session number.

EBP, Evidence Based Psychotherapy; CPT, Cognitive Processing Therapy; PE, Prolonged Exposure.

DS vs. D, Double-Single compared to Double coding; DS vs. C, Double-Single compared to Consensus coding; C vs. D, Consensus compared to Double coding.

### Interview procedures

To contextualize and explain variation in adherence and modifications, we conducted brief 30–45- min semi-structured interviews with a purposive sample of study therapists selected to ensure representation of CPT and PE therapists from different facilities, who varied in terms of adherence and outcomes that were the focus of the parent study. To obtain our sample of 32 therapists (15 CPT, 4 PE, 13 CPT, and PE therapists), we contacted 56 of the 182 therapists in the parent study first through email and then by telephone. Of the 56, 32 were interviewed. The interview asked about the use of the specific modifications included in FRAME. A full description of the interview process and methods will be presented elsewhere. In keeping with methods used for rapid analysis of qualitative data (12), the interview team jointly created detailed post-interview logs that detailed the main themes and new areas for further inquiry. For this paper, as a form of triangulation, we reviewed the interview logs from all 32 interviews to determine whether therapists were aware of using protocol modifications included in the FRAME (1).

### Statistical analysis

We calculated summary statistics describing protocol adherence and use of modifications at the session level, patient level, and provider level. At the session level, we assessed percentage protocol adherence, use of individual modifications, and number of modifications. At the patient level, we summarized individual patient measures of the overall percentage adherence across all attended sessions; as well as the presence, number, and proportion of sessions with each type of modification and of any modification. At the provider level, we summarized the average and variance in the percentage adherence of the providers' patients, the proportion of their patients with any modification and with each specific type of modification and any modification, the average and variance in the proportion of their patient's sessions with each modification and any modification, and the average and variance in the average number of modifications per session.

We then assessed the proportions of variance in session level adherence and modifications that can be attributed to differences between patients and differences between therapists rather than simple session to session variation. To accomplish this, we used multilevel modeling with patients nested within therapists, to partition the total variance in adherence and modifications into therapist and patient levels. The methodology for multilevel modeling is described elsewhere [e.g. (13)] and has been applied to the study of therapist effects on psychotherapy outcomes [e.g. (14)]. We fit random effects logistic regressions that included random effects for patients and for therapists, each assumed to follow normal distributions with mean zero and unspecified variance for the patient effects and unspecified variance for the therapist effects. Likelihood ratio tests were used to assess the significance of the respective random effects. We estimated the proportion of variance in the log odds for an outcome at the therapist and patient level using the ratio of the respective variance component estimate to the sum of the variance for the therapist effects, the patient effects, and the variance for the logistic distribution (as a measure of session variance conditional on the therapist and patient random effects).

We also examined associations between adherence and modifications in sessions and the type of rating employed to assess if the amounts of modifications and level of adherence played a role in the type of review that was needed for assessing these measures. We compared the patient level measures of modifications and adherence between the groups of patients with Double-Single, Double, and Consensus coding, using logistic regression likelihood ratio and Wald chi-squared tests and Kruskal-Wallis ranked score analysis of variance chi-squared tests. We fit a similar logistic regression model with random effects for therapists to assess the proportion of variance attributable to therapists in the use of Double-Single rated coding of modifications and adherence measures for a patient's course of therapy. We repeated this analysis for Consensus coding of adherence and modifications.

## Results

See Tables 2, 3 for therapist and patient characteristics. As hypothesized, modifications to the content of therapy sessions were common (Table 1). All therapists made at least one modification with at least one of their patients, and the majority (95%) of the patients in the sample had at least one modification throughout the course of their episode of care. This result is consistent with interview data that found that all interviewed therapists (*n* = 32) acknowledged making modifications to the treatment protocols.

**Table 2:** Characteristics CPT and PE therapists (*n* = 182).

**Characteristic**	***n* (%)**
**Sex**	
Female	96 (52.7)
Male	32 (17.6)
Missing	54 (29.7)
**Race**	
White	159 (87.4)
African American	6 (3.3)
Asian American	5 (2.7)
Multiracial	3 (1.6)
Other	2 (1.1)
Missing	7 (3.8)
**Hispanic/Latinx identity**	
Yes	173 (95.1)
No	4 (2.2)
Missing	5 (2.7)
**Professional discipline**	
Psychologist	76 (41.8)
Social Worker	100 (54.9)
Counselor	6 (3.3)
**Clinic role**	
Clinic leader	25 (13.7)
Staff	157 (86.3)
**Years in current clinic**	
< 1 year	7 (3.8)
1–5 years	107 (58.8)
6–10 years	46 (25.3)
> 11 years	22 (12.1)
**Preferred theoretical orientation**	
Behavioral or cognitive behavioral	138 (75.8)
Interpersonal	3 (1.6)
Psychodynamic	8 (4.4)
Humanistic	3 (1.6)
Eclectic	27 (14.8)
	3 (1.6)

**Table 3:** Characteristics of veterans whose CPT and PE sessions were rated for adherence and modifications (*N* = 1,257).

**Age (years)**	***M* = 46.78** **(*SD* = 14.00)**	**Range = 21–87**
	***N***	**%**
Female	300	23.87
Male	957	76.13
**Race**		
African American/Black	275	21.88
Native American	10	0.80
Hawaiian Pacific Islander	19	1.51
Asian	14	1.11
White	850	67.62
Unknown	78	6.21
Hispanic ethnicity	110	8.75
Not Hispanic	1,101	87.59
Unknown	46	3.66
**Current marital status**		
Married or partnered	845	67.28
Divorced or separated	254	20.22
Widowed	19	1.51
Single/never married	96	7.64
Unclear	43	3.42
**Education**		
Less than high school	6	0.48
High school or GED	256	20.46
Some college or trade school	371	29.66
College	193	15.47
> College	95	7.59
Unclear	336	26.73
**Employment status**		
Employed outside the home	606	48.36
Not employed outside the home	554	44.22
Unclear	93	7.42
Enrolled in educational program	145	11.85
**Military service era**		
OEF/OIF/OND	481	38.27
Persian Gulf	467	37.15
Post-Vietnam	118	9.39
Vietnam	181	14.40
Korean War	4	0.32
Other	6	0.48
**Branch of military service**		
Army	697	55.54
Marines	191	15.22
Navy	208	16.57
Air Force	152	12.11
Coast Guard or unknown	9	0.72
**Index trauma for CPT or PE**		
Combat	664	52.95
Military sexual trauma	223	17.78
Other sexual trauma	48	3.83
Other trauma type	271	21.61
Multiple sources	48	3.83

Demographic variable names reflect variable names in the VA medical records.

At a session level, modifications were not frequent. Certain modifications were more common than others, occurring in at least 10% of the sessions, including removing, changing the length of sessions from what is specified in the original protocols, repeating, and spreading.

As shown in Table 4 and consistent with our second hypothesis, both therapists and patients contributed to the variation in modifications identified by the raters. However, the amount each contributed varied by the types of modification, ranging from 1 to 36% of the variance in different modifications for patients and 2–28% for therapists. For example, patients contributed more substantially to the total variation in tailoring and in integration of another treatment into the protocol than did therapists. Patients also contributed to the decision to switch CPT type among those who received CPT (e.g., if a patient did not complete a written account of the trauma as planned, therapists changed to the version without a trauma account rather than re-assigning it), whereas therapists did not. However, therapists contributed to variation in repeating and removing session elements, whereas patients did not. Both contributed to spreading session content over multiple sessions and lengthening session time, although a somewhat higher proportion of variance was attributed to therapists. Both contributed to the variance found in drift.

**Table 4:** Estimated variance components for random effects for CPT and PE.

**Modification type**		**Estimate**	**SE**	**LRT p-value**	**Proportion of variance**
**Tailoring/Tweaking**					
Therapist effects		0.123	0.186	0.241	0.025
Patient effects		1.506	0.288	< 0.0001	0.306
**Switching CPT type**					
Therapist effects		0.247	0.400	0.259	0.052
Patient effects		1.194	0.470	0.002	0.252
**Integrating another treatment**					
Therapist effects		0.429	0.393	0.116	0.090
Patient effects		1.028	0.543	0.017	0.217
**Session lengthening/extending**					
Therapist effects		1.612	0.314	< 0.0001	0.261
Patient effects		1.276	0.187	< 0.0001	0.207
**Protocol lengthening/extending**					
Therapist effects		1.287	0.445	< 0.0001	0.281
Patient effects		NA (Scored at patient level across all sessions)			
**Session shortening/condensing**					
Therapist effects		0.498	0.102	< 0.0001	0.084
Patient effects		2.116	0.133	< 0.0001	0.358
**Repeating**					
Therapist effects		0.671	0.122	< 0.0001	0.169
Patient effects		0.017	0.058	0.380	0.004
**Reordering**					
NA (very rare event)					
**Spreading**					
Therapist effects		0.479	0.115	< 0.0001	0.116
Patient effects		0.367	0.097	< 0.0001	0.089
**Drift**					
Therapist effects		0.433	0.159	0.0002	0.098
Patient effects		0.698	0.218	< 0.0001	0.159
**Removing**					
Therapist effects		0.580	0.088	< 0.0001	0.148
Patient effects		0.040	0.039	0.133	0.010

^a^All modifications except protocol extending were based on EBP sessions 1 through 7.

CPT, Cognitive Processing Therapy; PE, Prolonged Exposure.

As expected, notes with low adherence were more challenging for raters, necessitating consensus coding to achieve consensus. Sessions that were Consensus coded had average adherence scores of.84 (*sd* = 0.14) compared to those Double coded entirely or at the session level, 0.87 (*sd* = 0.14) and 0.90 (*sd* = 0.13), respectively (see Table 5). As shown in Tables 4 and 5, the degree to which modifications were associated with different rating strategies differed by modification type. Spreading content from a single session across multiple sessions was present in 48% of the Consensus coded sessions, compared to 26% of the Double-Single coded and 20% of the completely Double coded sessions (*p* < 0.0002). Similarly, drift was found in 25% of the Consensus sessions, as compared to 14% of the Double coded and 10% of the Double-Single coded sessions. Repeating was also significantly more common among Consensus coded sessions (65%) compared to Double-Single (35%; *p* < 0.0001) or Double coded sessions (31%; *p* < 0.0001), which also differed significantly from one another (*p* = 0.019). Reordering of sessions was only found among Consensus rated sessions (1.5%). Tailoring was more consistent across coding methods at 12% for both Double and Consensus coding, although Double-Single coded sessions had a slightly but significantly lower proportion at 10% (*p* = 0.045). Shortening session length was more commonly found among Double coded sessions, although compressing session content from multiple sessions into a single session was more commonly found among Consensus coded sessions (15%; *p* < 0.0001 vs. 2% Double and 3% Double-Single coded sessions, which were significantly different from one another, *p* < 0.0024).

**Table 5:** Differences in modifications detected in different rating methods.

	**Double-Single**	**Consensus**	**Double**	**Overall X^2^**	**Pairwise comparison**	**X^2^**
	**(*N* = 451)**	**(*N* = 428)**	**(*N* = 378)**	***P*-value**		***P*-value**
	***n* (%)**	***n* (%)**	***n* (%)**			
Any modification	296 (66%)	357 (83%)	334 (88%)	*X*^2^ = 71.0, *p* < 0.0001	DS vs. D	< 0.0001
					DS vs. C	< 0.0001
					C vs. D	0.0460
Overall adherence – Flexible rating	0.90 (0.13)	0.84 (0.14)	0.87 (0.14)	*X*^2^ = 66.7, *p* < 0.0001	DS vs. D	< 0.0001
					DS vs. C	< 0.0001
					C vs. D	< 0.0001

DS vS. D, Double-Single compared to Double coding; DS vS. C, Double-Single compared to Consensus coding; C vS. D, Consensus compared to Double coding.

### Sources of rater disagreement using templated notes

As indicated in Table 4, there was higher adherence and there were fewer modifications in Double-Single coded sessions (approximately one-third of the sessions). However, Consensus coding was needed for about one third of patients, when raters found the notes to be confusing. As shown in Table 5 the “Consensus Rated” sessions had more modifications, suggesting that modifications contributed to difficulty rating notes.

Rater notes on the reasons for Consensus coding indicated that Consensus coding was needed when therapists repeated some but not all the essential elements from a specific session in a subsequent session. Based on their review of the documentation patterns, the raters observed that repetition of some but not all session elements occurred when patients did not complete assignments, arrived late so that the session was shortened or experienced technology problems. Some therapists wrote in the note that they were reintroducing material from prior sessions because the patient did not understand some of the already covered material. Another challenge in coding templates occurred when the documentation reflected discussion of current distressing life events rather than CPT or PE elements but also covered some session elements. Coders would need to review subsequent sessions to determine what session number to assign to the session with drift, and subsequent sessions where prescribed elements from the prior session as well as the expected material from the next session in the protocol that was covered. Another theme identified in the raters' notes was that it was difficult to use the templates to determine whether a therapy ending was planned. The documentation for a patient would stop at a certain point without it being clear as to the reason or the plan. The reason may have been that patients and therapists did not always know if a course of an EBP was going to continue when there was a break in treatment. This added burden to the rating process as raters would continue to look for templated and untemplated notes to know whether the therapy had ended. Finally, a modification that wasn't adequately described in the FRAME (1), which may warrant a new code for highly structured interventions, was the blending or combining of elements from multiple sessions. When this occurred, it became difficult for raters to code for adherence to a specific session or to use an existing modification code.

The raters also noted that therapists were adapting the templates in multiple ways. For example, at times they selected a template for a specific session number but either deleted the checklists for session elements or wrote in the notes section what they actually did that did not align with the prescribed elements for that session. They also often included elements that were intended for a specific session in earlier or later sessions. The patterns of modifications and adherence suggested that key protocol elements were typically completed over the course of the protocol, but not necessarily in the session that was specified in the treatment manuals.

## Discussion

We evaluated a method of identifying modifications to EBPs through clinical documentation. Consistent with prior research (5, 6, 9), we found that modifications are common during the use of EBPs for PTSD. While they do not occur in every session, over the course of the treatment protocol, most patients experience at least one modification, and all therapists modify the protocol in some ways. Our application of multilevel modeling to quantify patient and therapist contributions to variation in adherence and modifications represents a methodological advance to this area. We found that a substantial proportion of variance for some modifications was accounted for by patients (e.g., tailoring the protocol, integration of other treatments), while others appeared to be more driven by therapists. In fact, the magnitude of therapist contribution for many modifications is relatively large compared with previous studies of therapist effects on outcomes such as dropout and symptom change (15–17).

These findings have important implications for the refinement of treatment protocols and for therapist training. For example, understanding the patient characteristics associated with integration of other treatment elements and tailoring can provide treatment developers with information they can use to update treatment protocols and training materials to include guidance about providing treatment to individuals with those characteristics. Additionally, therapist-driven modifications such as repeating and removing elements may be addressed through training and fidelity support if they are not found to lead to better outcomes. While this study did not assess outcomes in relation to modifications, prior research indicated that modifications that were consistent with the protocol were associated with increased symptom change (9). A recent qualitative study further suggested that rigid protocol adherence was associated with treatment dropout, whereas more flexible and patient-centered application of EBPs for PTSD were associated with treatment completion (17). Thus, training on how to maintain flexibility while still ensuring that patients experience essential treatment components may be particularly helpful.

Our findings, in conjunction with prior research, also suggested that while it is feasible to use clinical documentation to track modifications, there are also challenges with using templates that are structured around a specific protocol. First, therapists documented their adherence and modifications inconsistently, and this necessitated careful inspection of the notes and consensus rating. Raters indicated that with the current templates, it was sometimes difficult to determine which template session number should be used. Existing guidance for use of templates (i.e., where one template is assigned to each type of session per protocol) made it challenging to evaluate adherence and modifications when therapists brought in content from prior sessions and did not cover all the prescribed material for the specified session. It was also challenging to evaluate adherence and modifications when therapists included information about current events and stressors in their notes, but it was unclear whether they were addressed within the EBP framework or through the use of different therapeutic strategies. While it was possible to achieve good rater agreement, consistent with previous studies [e.g. (6)] it was necessary to review the full episode of care to understand the types of modifications that occurred.

In light of the challenges and patterns of modifications we identified, we recommend a number of considerations when designing templates that can be used to assess adherence and modifications. Given how frequently therapists did not complete all required session elements and instead spread across multiple sessions, combined/blended session elements, or drifted to address emerging clinical issues, we recommend development of flexible templates that can more easily reflect what occurs in sessions. We recommend developing a single checklist of all required elements of structured protocols in a template that can be used across the entire protocol. A notation by each item indicating the session when it is expected to occur could still be included to support and guide fidelity, but the suggested approach would also allow a full reflection of the ways in which therapists provide the treatment in routine care. Anchoring adherence assessment to core elements, regardless of when they occur in the protocol, allows therapists greater ability to represent what actually occurred in their sessions. It may also allow for better detection of modifications such as repeating elements, changing the ordering or timing of specific elements, and spreading across multiple sessions. Including “yes,” “no,” and “partially” options for each core protocol element would also provide for more accurate representation of what occurred. Items to reflect decisions to terminate the protocol early, along with why, how, and by whom the decision was made could also be included to make the clinical decision-making process more transparent.

Additionally, items could be added to templates to reflect common modifications and reasons for modification. Embedding checklists of the possible types of modifications into templates would allow therapists to quickly document the changes that they made and why (e.g., patient was late, telehealth technical difficulties, emergent life stressors, or clinical issues that warrant attention). Encouraging providers to include rationale for modification and making space for the rationale in the documentation would allow therapists to describe the clinical judgements that they made in modifying the protocol. This would also facilitate coding of the rationale (1), especially if a checklist of reasons and goals for modifications are included in the in templates, perhaps as an optional item. Well-documented reasons for modification can also be fed back to treatment developers and training programs. These additions require a slight increase in the time required for documentation, but they would allow for much more clarity in understanding the clinical decision making process and may be more informative to other care providers who review the documentation to support patient care. Flexible clinical templates would also facilitate more effective training and consultation and/or audit and feedback systems. For both adherence and modification checklists, some training, or documentation support such as pop-up definitions or examples, may be necessary to ensure good reliability and efficiency.

While this is one of the first studies to evaluate the use of clinical documentation to assess modifications to EBPs and represents a larger sample of patients than previous research, some limitations are worth noting. First, we did not examine the association of modifications with other measures such as clinical outcomes, so whether different forms of modification should be encouraged or discouraged remains to be studied. Prior research suggests a relationship between some forms of modifications and clinical change (7, 9, 18), but whether the relationships vary based on setting or clinical population remains to be explored. However, we can conclude that therapists are delivering the treatments with more flexibility than protocols and templates allow and that modification is very common across CPT and PE protocols, as delivered in the VA. There were also some limitations to our method of examining different patterns of modifications based on coding strategy. While we saw different associations between modification types and coding strategies, our method can't determine whether the modifications led to the need for different rating strategies or if the rating strategies revealed different patterns of modifications, despite careful efforts to keep raters calibrated. Future research will need to be conducted in a manner that can rule out a potential method effect. Additionally, we did not compare ratings based on clinical documentation to observer-based ratings. Notably, though, we identified fewer modifications per session than found in a study that included observation of therapy sessions in a diverse community setting (9). Future research is needed to determine whether direct observation reveals a different number of modifications than clinical documentation.

In the current project, the sheer number of different types and combinations of modifications made it difficult to determine whether changes were fidelity-consistent (consistent with or explicitly allowed in the EBP protocol) or inconsistent, as this determination was highly context-dependent. Whether something is fidelity-consistent in EBPs may vary by the point in the protocol or be dependent on individual patient circumstances. Clear decision rules for what modifications are fidelity-consistent and inconsistent can help but may be nuanced unless a strict definition of adherence to core session elements within a specified period of time (or within a specific session) is adopted. Review and coding of some types of modifications by raters who have a good understanding of the treatment may be necessary if modifications need to be categorized as fidelity-consistent or inconsistent based on documentation. This has implications for the resources and personnel required for projects of this nature.

Despite these limitations and important next steps, our findings and description of methods for identifying modifications to EBP protocols based on clinical documentation suggest a path forward for using medical records to examine the types and outcomes for different forms of content modifications to psychotherapies. Our findings suggest that while using clinical documentation in medical records may be a pragmatic strategy in terms of reducing therapist burden, as currently designed, considerable time and effort is required to extract the information from medical record templates in the VA system. However, refinements like those suggested above, as well as the use of other approaches, such as training providers to clearly document their modifications in the free text sections of their notes, could advance efforts to understand how EBPs are modified in routine care settings. Research suggests that there is substantial room for improvement in terms of engagement of underrepresented populations (19, 20) and veterans in specialty care programs (17) and for optimizing CPT and PE patient outcomes (21–26) in large healthcare systems. With refinement, methods to understand modifications and fidelity to EBPs through medical record documentation can contribute to efforts to understand how to optimize EBPs for PTSD, and how to train and support providers as they use these interventions to treat their patients.

## Data availability statement

The datasets presented in this article are not readily available because they contain confidential medical records. Requests to access the datasets should be directed to Nina.Sayer@va.gov.

## Ethics statement

The studies involving human participants were reviewed and approved by Minneapolis VA Healthcare System Institutional Review Board. The therapist participants provided their written informed consent to participate in this study.

## Author contributions

SW, NS, and HL conceptualized the study and design, interpreted findings, and drafted the manuscript. DN collaborated on the design, conducted the statistical analyses, and drafted the quantiative findings. RO and AK coded data and contributed to the design and the manuscript. All authors contributed to the article and approved the submitted version.

## Funding

This research was supported by the Department of Veterans Affairs, Health Services Research and Development (HSR&D) grant (IIR 17-178) awarded to NS. The sponsor was not involved in any aspect of the study's design and conduct; data collection, management, analysis, or interpretation of data; or in the preparation, review or approval of the manuscript. The findings and conclusions presented in this manuscript are those of the authors and do not necessarily represent the views of the Department of Veterans Affairs or HSR&D.

## Conflict of interest

The authors declare that the research was conducted in the absence of any commercial or financial relationships that could be construed as a potential conflict of interest.

## Publisher's note

All claims expressed in this article are solely those of the authors and do not necessarily represent those of their affiliated organizations, or those of the publisher, the editors and the reviewers. Any product that may be evaluated in this article, or claim that may be made by its manufacturer, is not guaranteed or endorsed by the publisher.
